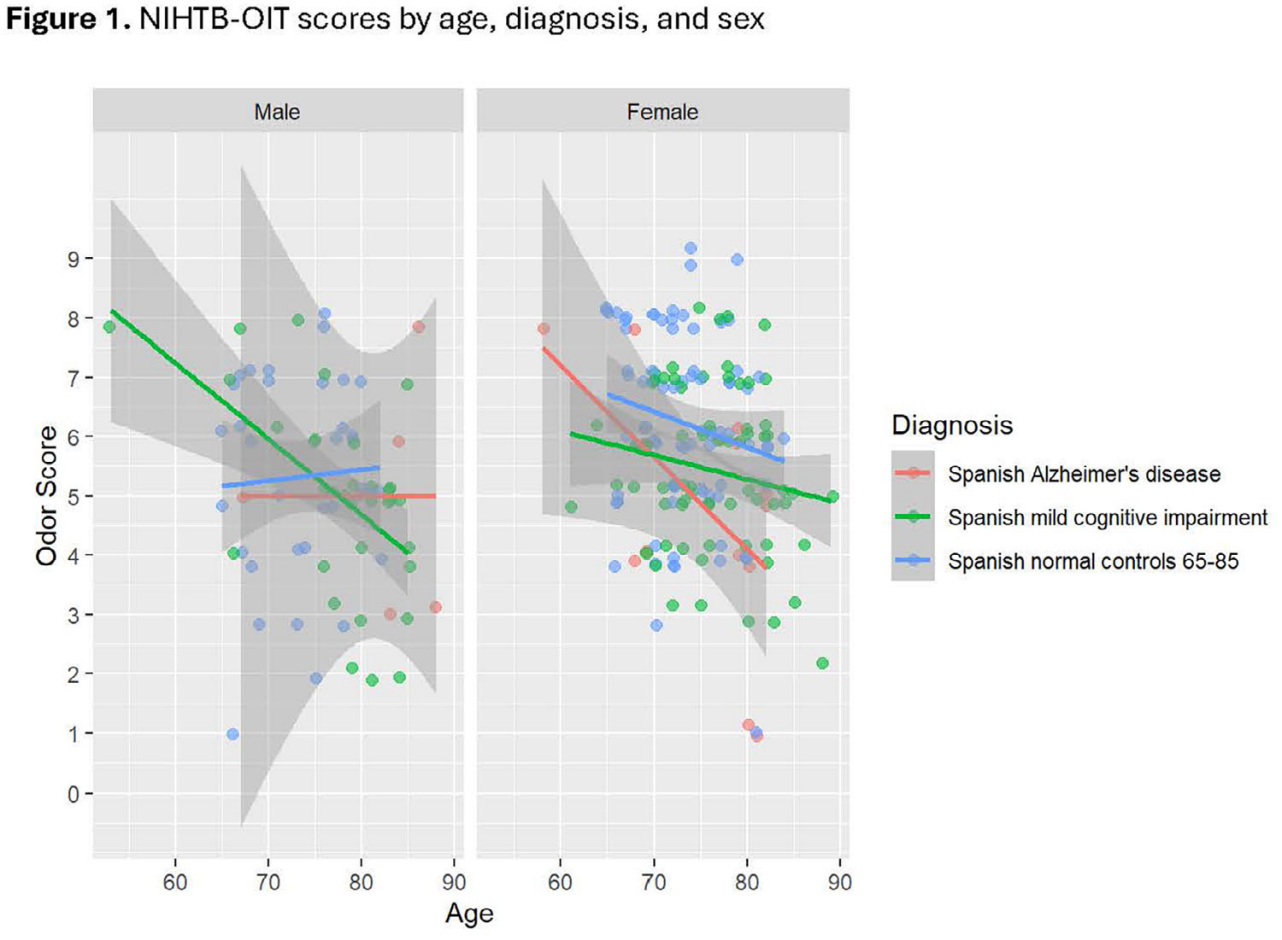# Evaluation of the NIH Toolbox Odor Identification Test across Healthy Agers, Mild Cognitive Impairment, and Alzheimer's Disease among Spanish‐speaking Older Adults: Results from the ARMADA Study

**DOI:** 10.1002/alz70857_102490

**Published:** 2025-12-25

**Authors:** Berivan Ece, Emily H Ho, Sandra Weintraub, Jennifer J. Manly, Richard C. Gershon

**Affiliations:** ^1^ Northwestern University Feinberg School of Medicine, Chicago, IL, USA; ^2^ Northwestern ADC Neuropathology Core, Northwestern University Feinberg School of Medicine, Chicago, IL, USA; ^3^ Department of Neurology, Columbia University, New York, NY, USA

## Abstract

**Background:**

Olfactory decline is correlated with cognitive decline in aging. The National Institutes of Health Toolbox Odor Identification Test (NIHTB‐OIT) is an established measure of olfaction ability. While it distinguishes differences in English‐speaking samples with varying cognitive abilities, its validation has not been completed for Spanish‐speaking and LatinX populations, who are far more likely to develop Alzheimer's than non‐Latinos. We evaluated the NIHTB‐OIT results from a cross‐sectional sample of Spanish‐speaking older adults.

**Methods:**

In the context of Advancing Reliable Measurement in Alzheimer's Disease and Cognitive Aging (ARMADA) Study, we evaluated 239 Spanish‐speaking adults aged 53 to 89: 115 normal controls (NC) (70.2% female; *M_age_* = 73.19, *SD_age_* = 4.99), 104 patients with mild cognitive impairment (MCI) diagnosis (68% female; *M_age_* = 76.56, *SD_age_* = 6.23), and 20 patients with Dementia of Alzheimer Type (DAT) diagnosis (70% female; *M_age_* = 76.95, *SD_age_* = 7.76). The NIHTB‐OIT was administered by trained examiners using scratch‐and‐sniff cards for odor identification. Participants’ performance is evaluated based on correct responses, with total raw scores ranging between 0 and 9. We analyzed the data using ANOVA for main effects and interactions, and Welch Two Sample t‐tests for sex differences.

**Results:**

Results indicated main effects of age (*F*(1, 230) = 13.27, *p* < .001, *ηp2* = .055), diagnosis (*F*(1, 230) = 3.59, *p* = .029, *ηp2* = .030), and sex (*F*(1, 230) = 6.21, *p* = .013, *ηp2* = .026). Older adults had lower scores on the NIHTB‐OIT. No pairwise comparison was significant across normal controls, MCI, and DAT groups. We observed sex differences only for the healthy agers (*p* < .001), with female participants (*M* = 6.24, *SD* = 1.56) having higher scores than males (*M* = 5.32, *SD* = 1.70). There was no significant interaction between age and diagnosis, while the interaction between age and cognitive status was significant. Finally, a three‐way interaction was observed between age, sex, and cognitive status (see Figure 1).

**Conclusions:**

Among Spanish‐speaking older adults, clinical groups displayed different trajectories in their performance on the NIHTB‐OIT by both cognitive status and gender, suggesting heterogeneous clinical manifestations in odor detection ability with increasing age.